# Genetic basis of calcifying cystic odontogenic tumors

**DOI:** 10.1371/journal.pone.0180224

**Published:** 2017-06-28

**Authors:** Akane Yukimori, Yu Oikawa, Kei-ichi Morita, Chi Thi Kim Nguyen, Hiroyuki Harada, Satoshi Yamaguchi, Kou Kayamori, Akira Yamaguchi, Tohru Ikeda, Kei Sakamoto

**Affiliations:** 1 Department of Oral Pathology, Graduate School of Medical and Dental Sciences, Tokyo Medical and Dental University, Tokyo, Japan; 2 Department of Oral and Maxillofacial Surgery, Graduate School of Medical and Dental Sciences, Tokyo Medical and Dental University, Tokyo, Japan; 3 Department of Maxillofacial Surgery, Graduate School of Medical and Dental Sciences, Tokyo Medical and Dental University, Tokyo, Japan; 4 Department of Bioresource Research Center, Tokyo Medical and Dental University, Tokyo, Japan; 5 Department of Oral Health Science Center, Tokyo Dental College, Tokyo, Japan; Peter MacCallum Cancer Centre, AUSTRALIA

## Abstract

Calcifying cystic odontogenic tumors (CCOTs) are benign cystic tumors that form abnormally keratinized ghost cells. Mutations in *CTNNB1*, which encodes beta-catenin, have been implicated in the development of these tumors, but a causal relationship has not been definitively established. Thus, mutational hot spots in 50 cancer genes were examined by targeted next-generation sequencing in 11 samples of CCOT. Mutations in *CTNNB1*, but not in other genes, were observed in 10 of 11 cases. These mutations constitutively activate beta-catenin signaling by abolishing the phosphorylation sites Asp32, Ser33, or Ser37, and are similar to those reported in pilomatrixoma and adamantinomatous craniopharyngioma. In contrast, *BRAF* or *NRAS* mutations were observed in 12 and two control samples of ameloblastoma, respectively. In HEK293 cells, overexpression of mutated CTNNB1 also upregulated hair keratin, a marker of ghost cells. Furthermore, ghost cells were present in two cases of ameloblastoma with *BRAF* and *CTNNB1* mutations, indicating that ghost cells form due to mutations in *CTNNB1*. The data suggest that mutations in *CTNNB1* are the major driver mutations of CCOT, and that CCOT is the genetic analog of pilomatrixoma and adamantinomatous craniopharyngioma in odontogenic tissue.

## Introduction

Odontogenic tumors develop in tooth-forming tissues in the jaw, or, rarely, in the gingiva. These tumors form as a wide range of lesions with diverse histological characteristics [1], highlighting the complexity of tooth morphogenesis and formation. Accordingly, the tumors are classified based on tissue of origin and histological features. For example, ameloblastoma, the most common odontogenic epithelial tumor, consists of tumor nests that resemble enamel-forming organs, but do not differentiate further to deposit enamel. Ameloblastoma is regarded as a true neoplasm, and is characterized by persistent and local infiltration into surrounding tissue. In contrast, tumors such as odontomas show only minor defects in tooth formation, and are thought to be hamartomatous lesions.

On the other hand, calcifying cystic odontogenic tumors (CCOT) are unique lesions that account for only 1–2% of odontogenic tumors. These tumors are characterized by cystic proliferation of odontogenic epithelium, and occasionally resemble ameloblastoma [1], although some are solid and present mixed histological features [1, 2]. The most prominent and defining microscopic feature is the formation of ghost cells, which are pale, swollen, and encapsulated, but devoid of nuclei. These cells are thought to form as a consequence of abnormal keratinization when tumor cells acquire trichogenic potential [3]. Several ways of subclassifying CCOT have been proposed [2, 4]. For example, Praetorius and coworkers [5] classified these tumors as cysts (Type I) or neoplasms (Type II). Cysts are unilocular, often associated with odontoma or an unerupted tooth, and are further subtyped as simple unicystic (Type IA), odontoma-producing (Type IB), or ameloblastomatous proliferating (Type IC). These cystic lesions are only weakly neoplastic. These lesions were termed calcifying odontogenic cysts in the 1971- and 1992-editions of the WHO histological typing of odontogenic tumors, and then termed CCOTs in the 2005-edition. In contrast, solid Type II neoplasms tend to infiltrate connective tissue, form ameloblastoma-like tumor nests, and are also called dentinogenic ghost cell tumors in the 2005- and 2017-edition. In the latest 2017-edition, the term ‘calcifying odontogenic cyst’ was adopted again, with CCOT listed as a synonym, and the entity was described in the category of cyst, and is not accounted as a tumor. The fluctuation of terminology reflects the lack of precise knowledge on their pathogenesis, as well as the overlapping definitions of tumors and cysts.

Recently, several mutations in genes along the Ras-BRAF pathway were identified by next-generation sequencing to be frequently associated with ameloblastoma. In particular, BRAF mutations were found in 46–63% of ameloblastoma cases [6–8]. However, the genetic basis of CCOT has not been extensively investigated. In 2003, Sekine and colleagues found that *CTNNB1*, which encodes beta-catenin, is frequently mutated in CCOT [9]. This pioneering work provided evidence that CCOT is a neoplastic lesion caused by genetic mutations. Whether *CTNNB1* mutations are the sole cause of CCOT, or one of many, is unknown. Furthermore, it is unclear whether CCOT subtypes are genetically distinct. Hence, we investigated the genetic and molecular basis of CCOT in greater detail.

## Materials and methods

### Sample selection

Formalin-fixed paraffin-embedded specimens collected from 30 CCOT (Type IA: 9 cases, Type IB: 18 cases, Type IC: 3 cases) patients between 1996 and 2016 were retrieved from the archive of Tokyo Medical and Dental University Dental Hospital. We excluded decalcified specimens and specimens in which PCR of a positive control target sequence (D10S1267) failed, leaving 11 tissues available for further DNA analysis. Tissue specimens of 14 ameloblastoma cases (five maxillary, six mandibular solid/multicystic, and three extraosseous/peripheral) were also retrieved for comparison. All experimental procedures were approved by the ethics committee of the Faculty of Dentistry, Tokyo Medicaland Dental University (Registration No. 1228). Since archived tissue specimens were originally obtained for diagnostic purposes, the institutional ethics committee consented to waive the requirement for specific informed consent in accordance with amended Ethical Guidelines for Clinical Studies provided by Ministry of Health, Labor and Welfare of Japan (July 31, 2008). This research plan was disclosed in a poster format in the outpatient clinic of the oral surgery department to ensure that patients had the opportunity to decline the research use of their pathological samples, which substituted for written informed consent, and the ethics committee approved this consent procedure. The archived tissue specimens were anonymized and used for research.

### Histology

Specimens were sectioned at 4 μm, stained with hematoxylin and eosin, reviewed, and, if necessary, re-diagnosed according to the 2005 World Health Organization classification of odontogenic tumors [1] by three experienced oral pathologists (A.Y., K.K., and K.S.). We counted the number of tumor cells and total cells in three or more representative microscopic fields imaged at 200x. In specimens where the number of tumor cells greatly varied across fields, especially in CCOT specimens, the average count among different fields of the section was taken, and the tumor cell ratio was calculated. The tumor cell ratio represents the ratio of the number of tumor cells to the total number of cells in the tissue. The tumor cell ratio was rounded off in increments of 10% in cases where the ratio was above 10%.

### DNA isolation and target-capture DNA sequencing

DNA was extracted from 20 μm sections using QIAamp DNA FFPE Tissue Kit (Qiagen, Hilden, Germany). Library preparation was performed using Ion AmpliSeq Library Kit 2.0 and Ion AmpliSeq Cancer Hotspot Panel v2 (Thermo Fisher Scientific, Waltham, MA, USA). The panel target’s hotspot regions included more than 2800 COSMIC mutations of 50 cancer-related genes. After the library preparation, each amplicon library was quantified using the Agilent 2100 Bioanalyzer and Agilent High Sensitivity DNA Kit (Agilent Technologies, Santa Clara, CA, USA) and sequenced using the Ion Proton platform and Ion PI Chip (Thermo Fisher Scientific). The average read depths were approximately 1100.

Data were analyzed using Torrent Suite Software v4.2.191 (Thermo Fisher Scientific) and Ion Reporter Software v4.6 (Thermo Fisher Scientific). The read alignments were performed using the human reference genome hg19. Candidate pathogenic variants were filtered based on the number of reads in a target sequence and variant frequency in the total number of reads. Intronic, homogeneous, or synonymous variants were excluded. Mutations were analyzed using SIFT, PolyPhen, and Mutation Taster, and were considered relevant when scored as deleterious by at least two of these algorithms.

### PCR and direct sequencing

Target sequences were amplified with PrimeSTAR GXL DNA Polymerase (Takara Bio, Shiga, Japan) by initial denaturation at 96°C for 1 min, 35 cycles at 96°C for 10 s, 58°C for 15 s, and 68°C for 20 s, and final extension at 68°C for 4 min. PCR products were visualized by agarose gel electrophoresis, purified using FastGene Gel/PCR Extraction Kit (Nippon Genetics, Tokyo, Japan), and sequenced by BigDye terminator v3.1 (Life Technologies). PCR primer sequences are listed in Table 1.

**Table 1:** PCR primers used.

BRAF codon600	F	5'-AACACATTTCAAGCCCCAAA-3'
	R	5'-GCATCTCAGGGCCAAAAA-3'
MAP2K1 exon2	F	5'-GACTTGTGCTCCCCACTTTG-3'
	R	5'-GTCCCCAGGCTTCTAAGTACC-3'
MAP2K1 exon3	F	5'-TCATCCCTTCCTCCCTCTTT-3'
	R	5'-CTCTTAAGGCCATTGCTCCA-3'
CTNNB1 codon32/33/34/37	F	5'-CCCTGGCTATCATTCTGCTT-3'
	R	5'-CCTCAGGATTGCCTTTACCA-3'

### Immunohistochemistry

Specimens sectioned at 4 μm were deparaffinized with xylene, and rehydrated through graded ethanol and then with water. Specimens were then probed according to Table 2 using antibodies against BRAF Val600Glu (1:4000, Clone VE1, E19290, mouse monoclonal, Spring Bioscience, Pleasanton, CA, USA), beta-catenin (1:50, Clone beta-catenin-1, M3539, mouse monoclonal, Dako, Glostrup, Denmark), and hair cortex keratin (1:500, Clone AE13, sc-57012, mouse monoclonal, Santa Cruz Biotechnology, Santa Cruz, CA, USA), and finally stained with 3,3'-diaminobenzidine. Specimens probed with non-immune serum were used as negative control, and these were confirmed to be unstained in pilot experiments.

**Table 2:** Protocol for immunohistochemical staining.

	VE1	βCatenin	AE13
Thickness	4 μm	4 μm	4 μm
Antigen retrieval	pH8.5 EDTA	pH9.0 EDTA	pH6.0 citrate buffer
	15 minutes at 121°C autoclave	40 minutes at 97°C microwave	60 minutes at 80°C microwave
Peroxidase block	10 minutes	30 minutes	30 minutes
Non-specific protein block	15 minutes	20 minutes	omitted
Primary antibody	Spring Bioscience, Pleasanton, CA, USA	DAKO, Glostrup, Denmark	Santa Cruz Biotechnology, Santa Cruz, CA, USA
	×4000	×50	×500
	4°C overnight	room temperature 60 minutes	4°C overnight
Detection system	Novolink Polymer Detection Systems (Leica Biosystems, Nussloch, Germany)	VECTASTAIN Elite ABC Mouse IgG Kit (Vector laboratories, Burlingame, CA, USA)	Envision+ Dual link system-HRP (DAKO, Glostrup, Denmark)

### Cell culture

Experimental procedures were approved by the Genetically Modified Organisms Safety Committee of Tokyo Medical and Dental University (Registration No. 2015-042C). Human embryonic kidney 293 cells were obtained from RIKEN Bioresource Center (Tsukuba, Japan), and maintained in Dulbecco’s modified Eagle’s medium with 10% fetal bovine serum. Cells were then transfected using Polyethylenimine Max (Polysciences, Warrington, PA, USA) with human wild type CTNNB1, which was provided by Eric Fearon (Addgene plasmid # 16828) [10], or the CTNNB1 mutant BcatMutS33/S37.T41/S45, which was provided by David Rimm (Addgene plasmid # 24204).

### Western blot and immunocytochemistry

Total protein was extracted from cells using buffer with cOmplete Protease Inhibitor (Roche Diagnostics, Basel, Switzerland). Keratin extracted from hair using 5 M urea, 2.6 M thiourea, 50 mM Tris-HCl pH 7.4, and 2% CHAPS was used as positive control. Samples were analyzed by western blot as previously described [11], using primary antibodies against hair cortex keratin (Clone AE13, Santa Cruz Biotechnology), beta-catenin (Clone beta-Catenin-1 Dako), and GAPDH (Clone D16H11, Cell Signaling Technology, Danvers, MA, USA). Rabbit anti-mouse IgG conjugated to horseradish peroxidase (Thermo Fisher Scientific, Waltham, MA, USA), donkey anti-rabbit IgG conjugated to horseradish peroxidase (Thermo Fisher Scientific), and goat anti-rabbit IgG conjugated to Alexa Fluor 488 (Thermo Fisher Scientific) were used as secondary antibodies.

For immunocytochemistry, cells were fixed in methanol for 5 min, rinsed three times with phosphate-buffered saline, and probed for 2 h at room temperature in 1:500 primary antibody. Samples were then rinsed three times with phosphate-buffered saline, stained for 1 h at room temperature with 1:500 fluorescently labeled secondary antibody supplemented with 1:20,000 DAPI (Dojindo, Kumamoto, Japan), rinsed another three times with phosphate-buffered saline, and mounted in fluorescent mounting medium (Dako).

## Results

### CTNNB1 mutations in CCOT tissues

As PCR against microdissected tissues failed in pilot studies, DNA was extracted from whole specimens, which contained fibroblasts, lymphocytes, and other cells in addition to tumor cells. To assess the impact of this contamination, the ratio of tumor cells to total cells was determined by histology, and hot spots in 50 genes commonly associated with cancer were analyzed by targeted next-generation sequencing. These genes are listed in Table 3, and include all genes reported to be mutated in ameloblastomas, seven archived samples of which were sequenced for comparison.

**Table 3:** Target genes covered by the ION AmpliSeq Cancer Hot Panel v2.

ABL1	EZH2	JAK3	PTEN
AKT1	FBXW7	IDH2	PTPN11
ALK	FGFR1	KDR	RB1
APC	FGFR2	KIT	RET
ATM	FGFR3	KRAS	SMAD4
BRAF	FLT3	MET	SMARCB1
CDH1	GNA11	MLH1	SMO
CDKN2A	GNAS	MPL	SRC
CSF1R	GNAQ	NOTCH1	STK11
CTNNB1	HNF1A	NPM1	TP53
EGFR	HRAS	NRAS	VHL
ERBB2	IDH1	PDGFRA	
ERBB4	JAK2	PIK3CA	

**Fig 1:**
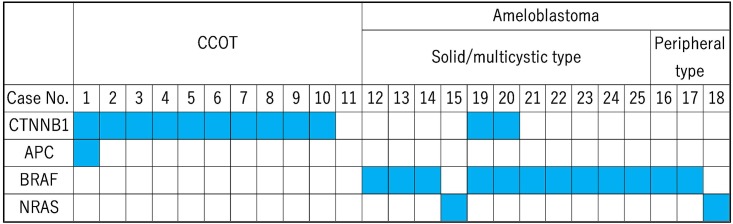
Summarized landscape of gene mutations in CCOT and ameloblastoma. Filled boxes indicate relevant gene mutations detected by next-generation (cases #1–18) and/or Sanger sequencing (cases #19–25). Note that only *CTNNB1*, *BRAF*, and *MEK2K1* hot spots were examined in cases #19–25, while hot spots in 50 cancer genes were examined in cases #1–18.

In CCOT, missense point mutations in *CTNNB1* were found in 10 of 11 cases (91%, Table 4, Fig 1). Eight of the 10 mutations altered Ser33 (six cases) and Ser37 (two cases) to phenylalanine (Ser33Phe and Ser37Phe) or cysteine (Ser33Cys). Of note, Ser33 and Ser37 are phosphorylation sites that inactivate Wnt/beta-catenin signaling via protein ubiquitination and degradation [12]. An Asp32Gly mutation was also found in two cases. While Asp32 is not a phosphorylation site, it is located in a degron. No other mutations were observed, except an additional *APC* mutation in one sample. In ameloblastoma samples, a Val600Glu mutation in *BRAF* was found in 5 of 7 cases, and a Gln61Arg mutation in *NRAS* was found in the other two cases (Table 4, Fig 1). The frequencies of all these mutations in the total reads were approximately half of the tumor cell ratio (S1 Fig), suggesting that they are somatic and monoallelic mutations.

**Table 4:** Case summaries.

	Case No.	Age (Year)	Sex	Location	Size (cm)	Subtype	Mutations in Next-generation sequencing	Sanger Sequencing			Staining			
								BRAF codon600	MAP2K1 exon2,3	CTNNB1 codon32,33,34,37	VE1	nuclear beta-catenin	Ghost cell	tumor cell ratio
Calcifying cystic odontogenic tumor	1	62	M	UR	2	IB	CTNBB1 p.Ser37Phe (c.110C>T), APC p.Pro1433Leu (c.4298C>T)	-	-	p.Ser37Phe	-	+	+	7
	2	10	M	UR	1	IA	CTNNB1 p.Ser37Phe (c.110C>T)	-	-	-	-	+	+	8
	3	66	F	UR	2	IB	CTNNB1 p.Ser33Cys (c.98C>G)	-	-	p.Ser33Cys	-	+	+	60
	4	41	M	LL	1.9	IB	CTNNB1 p.Ser33Cys (c.98C>G)	-	-	p.Ser33Cys	-	+	+	40
	5	21	F	LR	1.2	IB	CTNNB1 p.Ser33Cys (c.98C>G)	-	-	p.Ser33Cys	-	+	+	20
	6	70	F	UL	2	IA	CTNNB1 p.Ser33Cys (c.98C>G)	-	-	-	-	+	+	4
	7	38	F	UL	1	IA	CTNNB1 p.Ser33Phe (c.98C>T)	-	-	p.Ser33Phe	-	+	+	30
	8	15	M	UL	3	IB	CTNNB1 p.Ser33Phe (c.98C>T)	-	-	-	-	+	+	8
	9	13	M	LR	1.2	IA	CTNNB1 p.Asp32Gly (c.95A>G)	-	-	-	-	+	+	1
	10	72	M	LR	2.7	IC	CTNNB1 p.Asp32Gly (c95A>G)	-	-	p.Asp32Gly	-	+	+	50
	11	40	F	UR	0.5	IB	-	-	-	-	-	+	+	2
Ameloblastoma	12	37	M	UR	3.7		BRAF p.Val600Glu (c.1799T>A)	p.Val600Glu	-	-	+	-	-	70
	13	50	M	UL	2.5		BRAF p.Val600Glu (c.1799T>A)	-	-	-	+	-	-	10
	14	19	M	LL	5.4		BRAF p.Val600Glu (c.1799T>A)	p.Val600Glu	-	-	+	-	-	40
	15	77	F	LR	7.3		NRAS p.Gln61Arg (c.182A>G)	-	-	-	-	-	-	90
	16	61	M	LRg	1.1		BRAF p.Val600Glu (c.1799T>A)	p.Val600Glu	-	-	+	-	-	30
	17	79	M	LLg	1.5		BRAF p.Val600Glu (c.1799T>A)	p.Val600Glu	-	-	+	-	-	50
	18	67	M	ULg	1.8		NRAS p.Gln61Arg (c.182A>G)	-	-	-	-	-	-	70
	19	78	M	UL	4.3			p.Val600Glu	-	p.Ser37Cys	+	+	+	70
	20	37	M	LL	3.5			p.Val600Glu	-	p.Gly34Glu	+	+	+	50
	21	31	F	UL	2.5			p.Val600Glu	-	-	+	-	-	50
	22	64	M	UL	1			p.Val600Glu	-	-	+	-	-	10
	23	21	M	LR	4.5			p.Val600Glu	-	-	+	-	-	70
	24	71	M	LR	6.7			p.Val600Glu	-	-	+	-	-	50
	25	65	F	LR	2.3			p.Val600Glu	-	-	+	-	-	70

LL, lower jaw (mandible), left; LR, lower jaw, right; UL, upper jaw (maxilla), left; UR, upper jaw, right; LRg, mandibular gingiva (peripheral), right; LLg, mandibular gingiva(peripheral), left; ULg, maxillary gingiva (peripheral), left.

Size denotes the maximum diameter of the tumor. Tumor cell ratio denotes the ratio of the number of tumor cells to the total number of cells in the tissue.

In summary, CCOT is associated with characteristic missense mutations in *CTNNB1*, but not in other genes, regardless of the subtype.

### Sanger sequencing and immunohistochemistry

PCR and Sanger sequencing were used to confirm results from next-generation sequencing, and to test whether pathogenic *CTNNB1* and *BRAF* mutations are detectable by simpler laboratory methods. In particular, we sequenced PCR products containing *CTNNB1* codon 33–41 and BRAF codon 600. Exons 2 and 3 of *MAP2K1* were also sequenced as additional controls. *MAP2K1*, which encodes MEK1, was not analyzed by next-generation sequencing, and mutations in this gene were detected in Langerhans cell histiocytosis in the absence of *BRAF* mutations. We also tested an additional seven cases of ameloblastoma that were not analyzed by next-generation sequencing.

**Fig 2:**
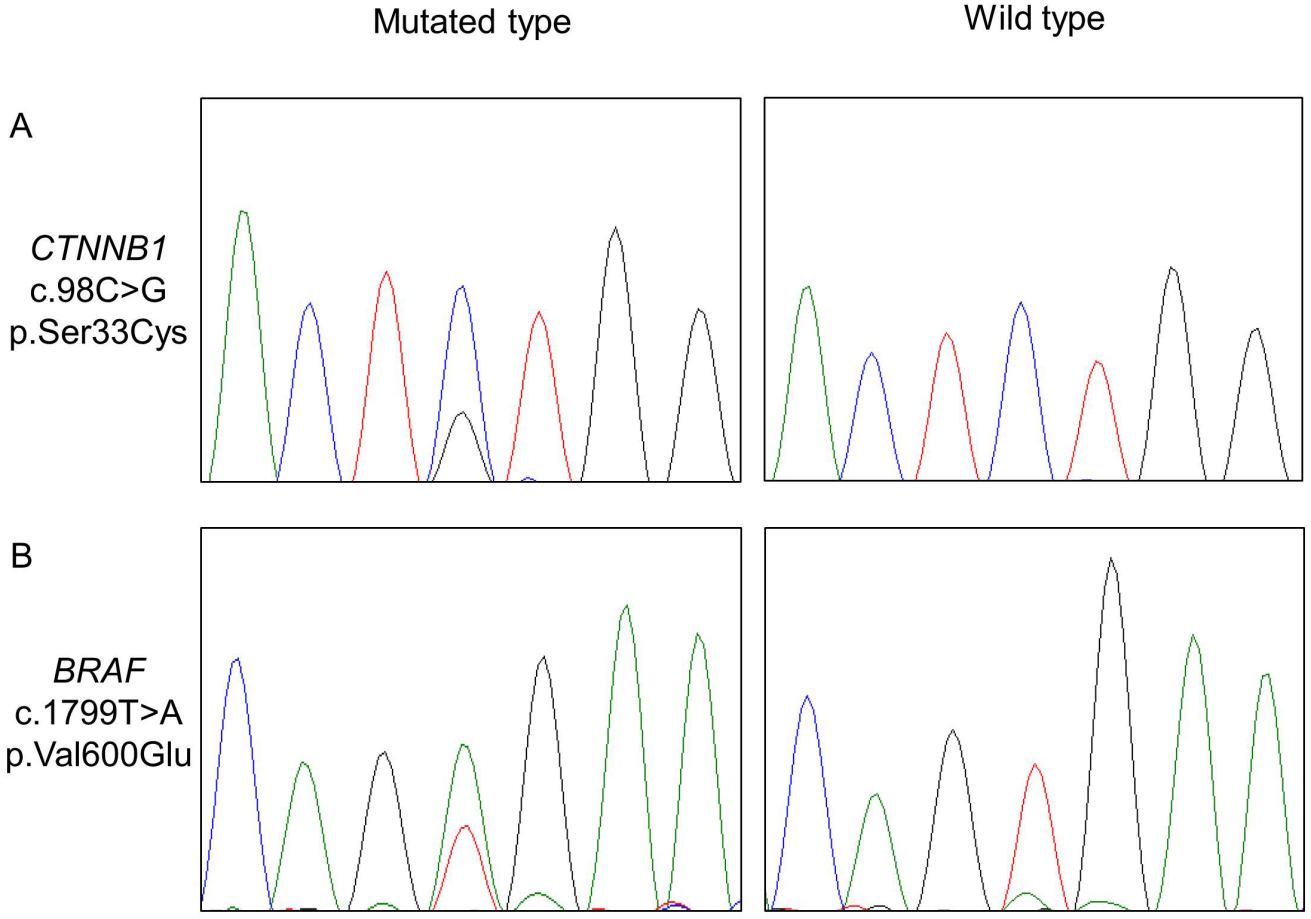
Electropherogram in CCOT and ameloblastoma. **A**, Electropherogram of a TCT>TGT substitution at c.98 in CTNNB1, resulting in a Ser33Cys missense mutation in case #4. **B**, Electropherogram of a GTG>GAG substitution at position c.1799 in BRAF, resulting in Val600Glu missense mutation in case #25. Guanine is indicated by a black line, cytosine is indicated by a blue line, adenine is indicated by a green line, and thymine is indicated by a red line.

*CTNNB1* mutations were detected by Sanger sequencing in six of 10 CCOT samples in which mutations were detected by next-generation sequencing (Table 4, Fig 2A). In cases #2, #6, #8, and #9, mutated bases were visible but were below the limit of detection, likely because the specimens contained small amounts of tumor cells, showing relatively low tumor cell ratios. The *BRAF* Val600Glu mutation was detected in 12 of 14 ameloblastomas, confirming the prevalence of this mutation (Table 4, Figs 1 and 2B). Thus, next-generation and direct sequencing collectively indicate that all 14 ameloblastomas harbor either *BRAF* Val600Glu or *NRAS* Gln61Arg mutations. Notably, *CTNNB1* Ser37Cys and *CTNNB1* Gly34Glu were found in addition to *BRAF* Val600Glu in ameloblastoma cases #19 and #20, respectively. However, mutations were not observed in *MAP2K1* exon 2 and 3.

**Fig 3:**
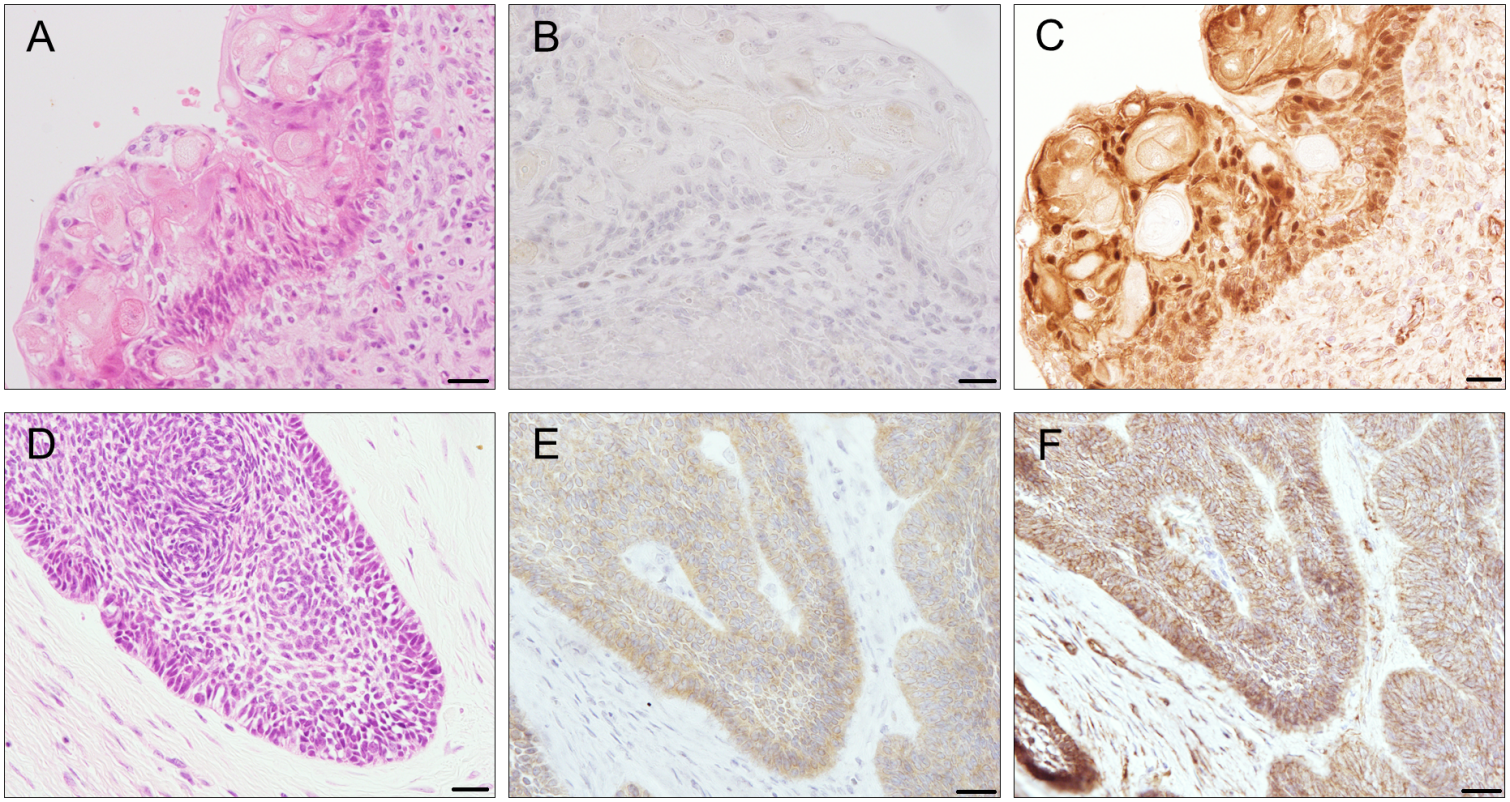
Photomicrographs of CCOT and ameloblastoma. **A, and D**, Representative photomicrographs of CCOT (case #6) and ameloblastoma (case #25) specimens stained with hematoxylin and eosin. **B, C, E, and F**, Immunostaining for (**B and E**) BRAF Val600Glu (clone name VE1) and (**C and F**) beta-catenin. Scale bars; 20 μm.

We then analyzed tissues by immunohistochemical staining for nuclear beta-catenin, a hallmark of active Wnt/beta-catenin signaling. Nuclear beta-catenin was observed in all cases of CCOT, although not in all tumor cells in a sample (Fig 3). In particular, tumor cells surrounding ghost cells tended to be enriched in nuclear CTNNB1. Ameloblastoma cases with *CTNNB1* mutations (cases #19 and #20) also accumulated nuclear beta-catenin. In contrast, beta-catenin was exclusively cytoplasmic in tumors with wild type *CTNNB1*, suggesting that pathogenic mutations in *CTNNB1* relocate beta-catenin to the nucleus. Finally, we confirmed by immunohistochemical staining that BRAF Val600Glu was expressed in the 12 ameloblastoma tissues with this mutation (Fig 3).

### *CTNNB1* mutations are associated with formation of ghost cells

**Fig 4:**
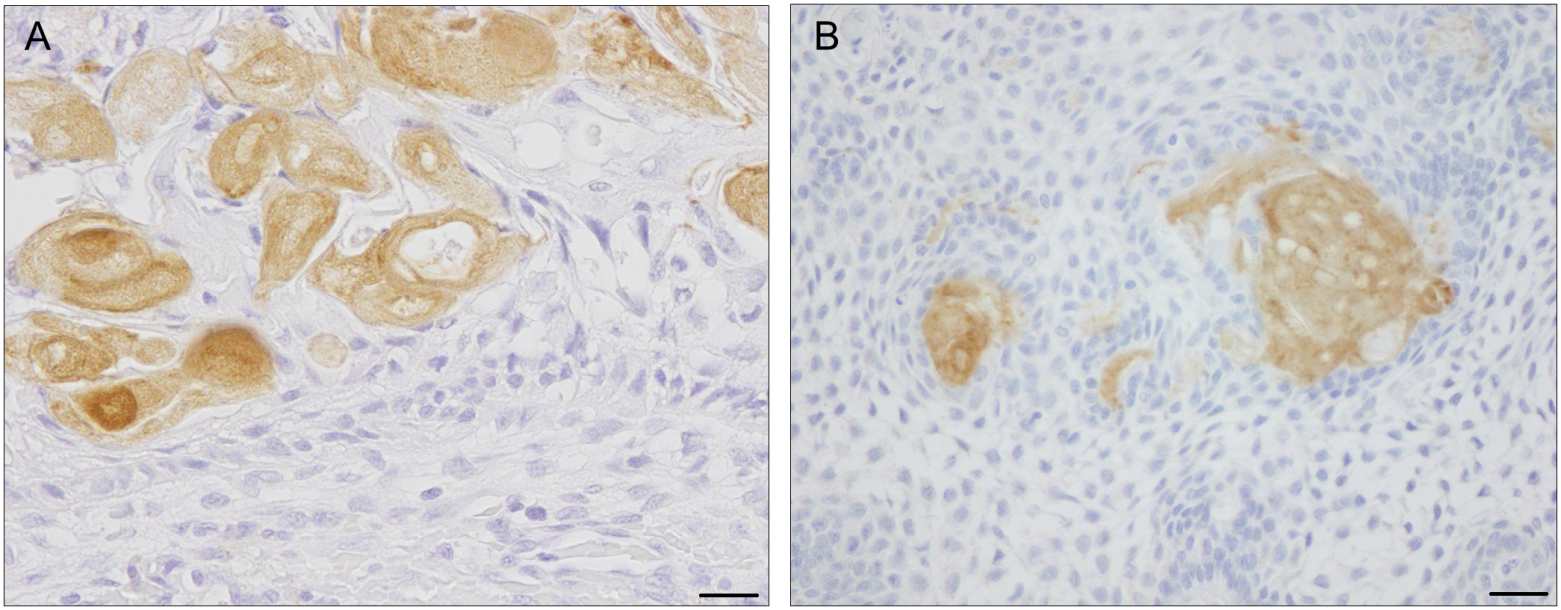
Photomicrographs of ghost cells in CCOT and ameloblastoma. **A, and B**, Representative photomicrographs of ghost cells immunostained for hair cortex keratin (clone name AE13) in (**A**) CCOT (case #6) and (**B**) ameloblastoma (case #20). Scale bars; 20 μm.

We hypothesized that *CTNNB1* mutations may drive the formation of characteristic ghost cells in CCOT. Thus, we closely examined cases of ameloblastoma with both *BRAF* and *CTNNB1* mutations (cases #19 and #20). In addition to histological features consistent with ameloblastoma, we observed in both cases a small number of ghost cells identifiable not only by the unique morphology, but also by the expression of hair keratin. Indeed, ghost cells in all cases of CCOT, as well as in ameloblastoma cases #19 and #20, specifically expressed hair keratin, as shown in Fig 4. In contrast, cells expressing hair keratin were not observed in ameloblastomas without *CTNNB1* mutations.

**Fig 5:**
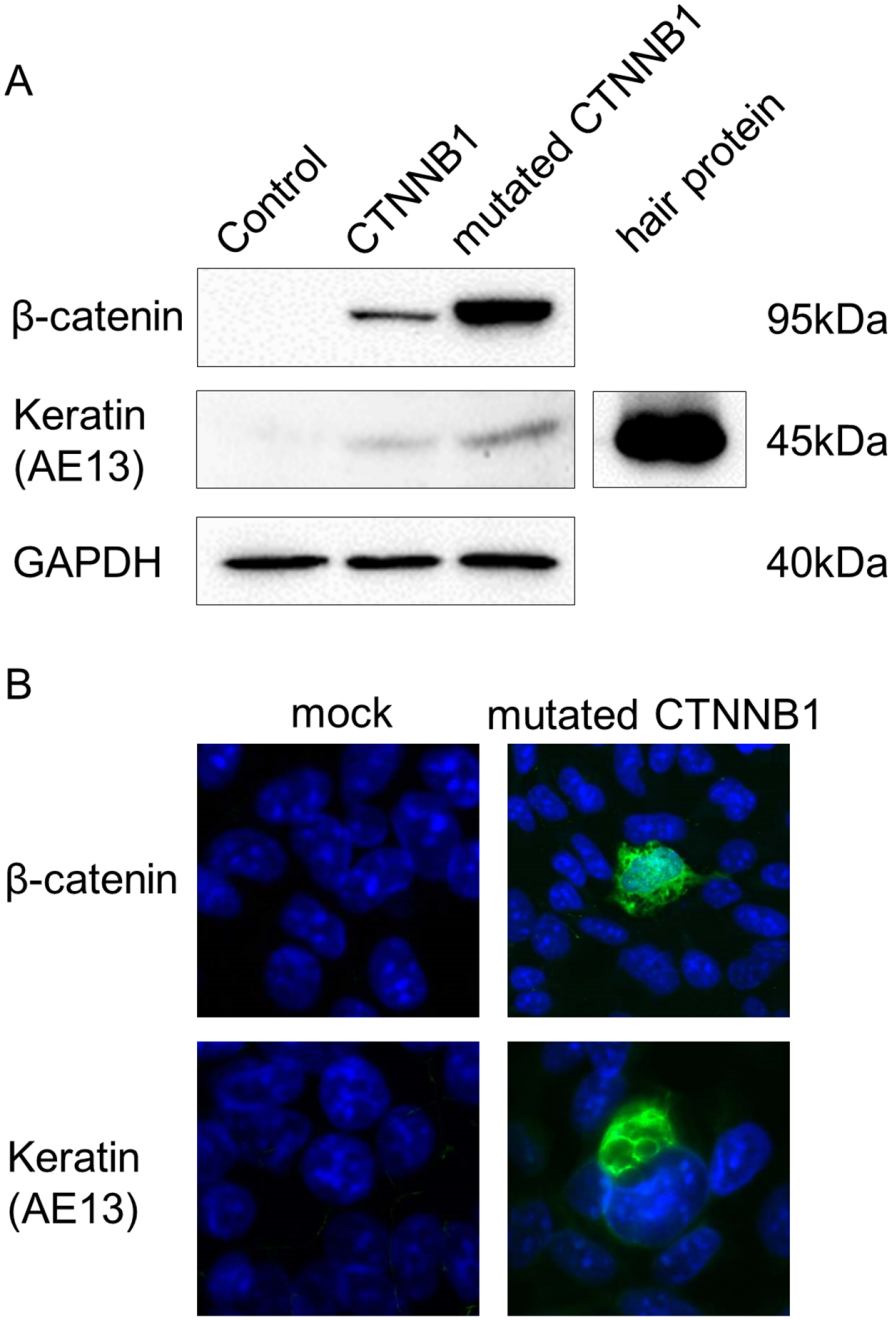
CTNNB1 induces hair keratin expression in human embryonic kidney 293 cells. **A**, Western blot for beta-catenin, hair keratin, and GAPDH. Cells were transfected with mock plasmid, CTNNB1, or mutant CTNNB1, and proteins were extracted 48 h after transfection. Protein extracted from a 1-mm fragment of hair was used as a positive control for hair keratin (clone name AE13). **B**, Immunofluorescent imaging of cytoplasmic and nuclear beta-catenin (green) in cells transfected with mutant CTNNB1, as well as expression of hair keratin (green) in DAPI-stained cells (blue). Original magnification 40x.

To further investigate the formation of ghost cells, human embryonic kidney 293 cells were transfected with a plasmid encoding *CTNNB1* with or without Ala substitutions of the phosphorylation sites Ser33, Ser37, Thr41, and Ser45. Western blot 48 h after transfection indicated that transfection with wild type or mutant *CTNNB1* upregulated expression of hair keratin (Fig 5A). Notably, the CTNNB1 mutant was more abundantly expressed than wild type, with expression proportional to that of hair keratin. Immunofluorescence staining confirmed that a few transfected cells expressed hair cortex keratin, although most cells did not, with mutant CTNNB1 accumulating in the nucleus as well as in the cytoplasm (Fig 5B). Collectively, these results suggest that CTNNB1 mutations that constitutively activate beta-catenin signaling also cause ectopic expression of hair keratin, and trigger the unusual trichogenic state of ghost cells.

## Discussion

In 10 of 11 archived CCOT samples, mutations were found exclusively in *CTNNB1*. Similar *CTNNB1* mutations have been reported in various cancers such as colorectal or lung adenocarcinoma, but at relatively low frequencies of less than 5% [13], suggesting that such mutations are not essential to many of those tumors. In contrast, the prevalence of somatic *CTNNB1* mutations in CCOT, as well as the absence of mutations in other genes, strongly suggests a causal relationship.

Sekine and coworkers [9] previously identified *CTNNB1* mutations in 9 of 11 CCOT cases (Asp32 (two cases), Ser33 (two cases), Gly34 (two cases), Ser37 (one case), and Tyr41 (two cases)). Recently, Sousa and colleagues [14] analyzed three cases of CCOT using the same cancer hot spot panel we used, and discovered a *CTNNB1* Ser33Phe mutation in two cases. In our cohort, Ser33 was the most frequently mutated (six of 11 cases, 55%), followed by Gly32 (two cases, 18%), and Ser37 (two cases, 18%). These mutations are similar to those detected in various tumors, including pilomatrixoma [15] and craniopharyngioma [16]. Pilomatrixoma is a skin tumor that develops from hair follicle matrix cells [17], while craniopharyngioma is an epithelial tumor that develops in the sellar region, and is subtyped by histopathology into adamantinomatous and papillary tumors [18]. Of note, pilomatrixoma and adamantinomatous craniopharyngioma consist of nests of basaloid cells with deeply eosinophilic tumor cells lacking nuclei and resembling ghost cells in CCOT. Indeed, the similarity of histological features in these tumors, despite different tissues of origin, indicates a common pathogenic mechanism, in which mutated CTNNB1 accumulates in the nucleus and elicits differentiation into hair [19–21]. Collectively, these results suggest that CCOT is the genetic analog of pilomatrixoma and adamantinomatous craniopharyngioma in the odontogenic tissue.

Phosphorylation of CTNNB1 Ser33, Ser37, Tyr41, and Ser45 via glycogen synthase kinase-3β (GSK-3β) leads to proteolytic degradation of CTNNB1. Activation of WNT signaling inhibits GSK-3β and stabilizes CTNNB1. This promotes nuclear accumulation of CTNNB1, and triggers transcriptional upregulation of downstream genes [22] to stimulate cell proliferation and inhibit apoptosis [12, 22]. Thus, mutations to these phosphorylation sites or to surrounding residues result in constitutively active beta-catenin signaling and tumor growth.

On the other hand, we found that *BRAF* Val600Glu appeared to be the only mutation in most ameloblastoma cases (86%, 12/14). Indeed, the mutation activates the MAPK pathway, promotes tumor progression [23], and is present in many tumors, including colorectal cancer [24], melanoma [25], and Langerhans cell histiocytosis [26]. Interestingly, papillary craniopharyngioma is also caused by *BRAF* Val600Glu instead of a *CTNNB1* mutation [27]. In our series of samples, *BRAF* Val600Glu was more frequent than that seen in previous studies, which reported frequencies of 46% (13/28) [8] and 62% (31/50) [7]. In ameloblastoma without *BRAF* Val600Glu, we found *NRAS* Gln61Arg, which is in agreement with a previous report showing that *RAS* mutations (including *NRAS* Gln61Arg) and *BRAF* Val600Glu are mutually exclusive [7]. Although *SMO* mutations have been identified in ameloblastoma in previous reports [7, 8], no *SMO* mutations were detected in our samples.

The data conclusively demonstrate that the odontogenic epithelial tumors CCOT and ameloblastoma are caused by the same panel of mutations that cause tumors in other tissues. However, unlike many malignant epithelial tumors with multiple and diverse genetic lesions, CCOT and ameloblastoma harbor mutations that are clonal and basically mutually exclusive, as is observed in adamantinomatous and papillary craniopharyngioma. Nevertheless, some cases of odontogenic tumors genetically overlap, such as ameloblastoma cases #19 and #20, in which *BRAF* and *CTNNB1* are present. These lesions were originally diagnosed as ameloblastoma because only a few ghost cells were observed in one or two tumor nests, but these cases might have been better diagnosed as dentinogenic ghost cell tumors to account for both genotype and phenotype. Further study is required for rendering consistency between the genotype and phenotype in diagnostic classification.

In conclusion, we demonstrated that most CCOT are neoplastic lesions due to mutations in *CTNNB1*. Although genetic analysis is useful to support a diagnosis of CCOT, the presence of ghost cells appears to be sufficient to identify an underlying genotype.

## Supporting information

S1 Fig Tumor cell ratio and frequency of the number of reads with the mutations. Blue: tumor cell ratio (number of tumor cells/number of cells in the tissue). The numbers of tumor cells and total cells in three or more representative microscopic fields imaged at 200x were counted. In specimens where the number of tumor cells greatly varied across fields, the estimated counts in each field were combined throughout the whole section to calculate the tumor cell ratio. The tumor cell ratio was rounded off in increments of 10% in cases where the ratio was above 10%. Orange: frequency of the number of reads with the mutation versus the total number of reads. Horizontal axis depicts case numbers.
(TIF)
