# Supplementary figures and images for: Genetic basis of calcifying cystic odontogenic tumors
Source: PLoS One. 2017 Jun 28;12(6):e0180224. doi: 10.1371/journal.pone.0180224 (PMC5489209; doi:10.1371/journal.pone.0180224)

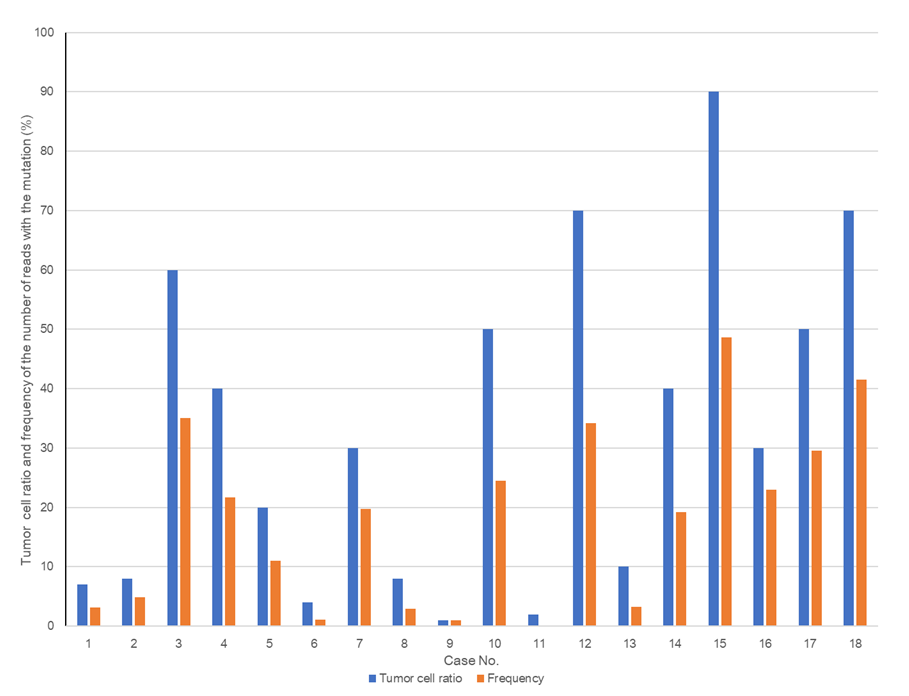

Supplement: S1 Fig — Blue: tumor cell ratio (number of tumor cells/number of cells in the tissue). The numbers of tumor cells and total cells in three or more representative microscopic fields imaged at 200x were counted. In specimens where the number of tumor cells greatly varied across fields, the estimated counts in each field were combined throughout the whole section to calculate the tumor cell ratio. The tumor cell ratio was rounded off in increments of 10% in cases where the ratio was above 10%. Orange: frequency of the number of reads with the mutation versus the total number of reads. Horizontal axis depicts case numbers. (TIF) [file pone.0180224.s001.tif]
